# Standardized GMP-compliant scalable production of human pancreas organoids

**DOI:** 10.1186/s13287-020-1585-2

**Published:** 2020-03-04

**Authors:** Marta Dossena, Roberta Piras, Alessandro Cherubini, Mario Barilani, Erica Dugnani, Francesca Salanitro, Till Moreth, Francesco Pampaloni, Lorenzo Piemonti, Lorenza Lazzari

**Affiliations:** 1 Laboratory of Regenerative Medicine - Cell Factory, Department of Transfusion Medicine and Hematology, Fondazione IRCCS Ca’ Granda Ospedale Maggiore Policlinico, Via F. Sforza 35, 20122 Milan, Italy; 2 Department of Clinical Sciences and Community Health, University of Milan, Milan, Italy; 3 IRCCS Ospedale San Raffaele, San Raffaele Diabetes Research Institute, Milan, Italy; 4 Physical Biology Group, Buchmann Institute for Molecular Life Sciences (BMLS), Goethe Universität Frankfurt am Main, Frankfurt am Main, Germany; 5 Vita-Salute San Raffaele University, Milan, Italy

## Abstract

**Background:**

Organoids are three-dimensional in vitro-grown cell clusters that recapitulate key features of native organs. In regenerative medicine, organoid technology represents a promising approach for the replacement of severely damaged organs, such as the pancreas in patients with type 1 diabetes. Isolation human pancreas organoids (hPOs) in chemically defined serum-free culture media would be a major milestone for this approach.

**Methods:**

Starting from discarded pancreatic tissues, we developed a large-scale process for obtaining clinically relevant quantities of undifferentiated organoids, obviating enzymatic digestion and operator-dependent pancreatic ducts picking steps. hPO identity was characterized by molecular and flow cytometry analysis.

**Results:**

This work demonstrates that it is possible to obtain a large-scale production of organoids. We introduced some innovations in the isolation, expansion, and freezing of hPOs from five donors. First of all, the choice of the starting material (islet-depleted pancreas) that allows obtaining a high quantity of hPOs at low passages. On the other hand, we introduced mechanical dissociation and we eliminated the picking step to exclude the operator-depending steps, without affecting the success of the culture (100% success rate). Another important improvement was to replace R-spondin-1 (Rspo1) conditioned medium with Rspo1 recombinant molecule to obtain a well-defined composition of the expansion medium. Finally, we implemented a GMP-compliant freezing protocol. hPOs showed exponential growth with diameter and area that increased three- and eight-fold in 7 days, respectively. Immunophenotypic profile and gene expression analysis revealed that hPOs were composed of ductal (82.33 ± 8.37%), acinar (2.80 ± 1.25%) cells, and pancreatic progenitors (5.81 ± 2.65%).

**Conclusion:**

This work represents a milestone for a GMP-compliance hPO production and, ultimately, their clinical application as a type 1 diabetes therapy.

## Introduction

Organoids are three-dimensional cellular structures generated from induced pluripotent stem cells, embryonic stem cells, or adult tissue-resident progenitor cells. They are valued for their capacity to self-organize into minimal biological units, which exhibit functionality and complexity similar to those of the tissue of origin. Organoids are an important bridge between two-dimensional cultures and in vivo models because they are more physiologically relevant than monolayer cell culture models while being far more amenable to manipulation of niche components, signaling pathways, and genome editing than in vivo models [1]. Since 2009, many protocols have been implemented for the culture of organoids isolated from various tissues, such as liver [2–5], pancreas [6–8], lung [9], gut [10–12], stomach [13, 14], and prostate [15]. Consequently, organoids can provide excellent model systems for a wide range of basic research studies, including expression profiling studies and analyses of rare cell lineages that are difficult to access in vivo [16]. In addition, organoids can be engrafted in vivo after transplantation for functionality assessment [17]. Furthermore, organoids generated from patients’ own tissues or induced pluripotent stem cells can be used to study rare diseases lacking an established animal model [18]. Indeed, human organoids are already being used to test and screen novel bioactive chemical compounds and in the development of personalized treatment regimens [19].

There is great interest in the potential use of organoids in regenerative medicine, especially for diseases involving severe organ damage but lacking a functional cell replacement cure, such as type 1 diabetes. In type 1 diabetes, which accounts for about 5% of diabetes cases worldwide, autoimmune activity destroys pancreatic β cells [20]. Insulin is a life-saving first-line treatment for type 1 diabetes, but it neither cures the disease nor prevents its long-term complications, such as heart attack, stroke, and peripheral vascular disease [21]. Currently, pancreas islet transplantation is the only therapeutic approach that restores a pool of functional β cells. Transplanted islets assume eloquent physiological control of blood glucose levels in a manner not achievable with insulin injections. Because islets have not been expanded successfully in vitro thus far, only fresh islets are used for transplantation [22]. However, there is a lack of suitable human islet donors. Indeed, the International Diabetes Federation estimates that 415 million people worldwide have diabetes, a number that is predicted to increase to 642 million by 2040 (http://www.diabetesatlas.org).

Human pancreas organoids (hPOs) may represent a suitable source of functional cells that, if therapeutic quantities are achieved, could be an effective treatment for type 1 diabetes. Importantly, undifferentiated hPOs recapitulate the phenotype of pancreas ductal epithelia in vitro, with a hollow spherical polarized cell monolayer enclosing a central lumen [23]. Furthermore, they can be cultured, expanded, and differentiated into endocrine lineage cells in vivo and in vitro [8, 23]. Currently, hPOs can be generated from primary pancreas tissue [3] or induced pluripotent stem cells [24]. Up to now, induced pluripotent stem cells are the only source that may guarantee large-scale production of pancreatic progenitors and differentiated derivatives [24]. However, adult endogenous pancreas stem cells are safer and raise less ethical issues than induced pluripotent stem cells, facilitating regulatory compliance.

In 2016, the European Community established support for a consortium formed by eight academic and industrial partners from six countries to develop tools and technologies for hPO-based type 1 diabetes therapy (www.lsfm4life.eu). The primary goal of this group is to conceive and implement a strategy for scaling-up undifferentiated hPO production to meet the massive type 1 diabetes clinical needs in accordance with good manufacturing practice (GMP) standards.

Although the development of hPOs from human pancreas biopsies has been demonstrated to be feasible [23], it is not yet possible to produce a clinically relevant number of hPOs for biobanking from a single biopsy. Thus, a larger quantity of starting material is needed to produce undifferentiated hPOs that may be subsequently differentiated after thawing for specific therapeutic applications. Therefore, the goal of this work was to establish a GMP-compliant protocol for the automated generation of biobank-appropriate, off-the-shelf and ready-to-use undifferentiated hPOs. This work is the first step in a process intended to lead to the timely entry of a promising new hPO-based allogeneic therapy for type 1 diabetes into clinical trials.

## Research design and methods

### Human adult pancreatic tissue

Human healthy pancreata were obtained from the Diabetes Research Institute, IRCCS Ospedale San Raffaele, Milan, Italy from multi-organ donors (Additional file 1: Table S1). The use of human specimens was approved by the Institutional Review Board. Human islets were isolated as previously described [25] at the Pancreatic Islet Processing Unit, a National Transplant Center accredited facility (IT000679, https://webgate.ec.europa.eu/eucoding/reports/te/index.xhtml). Acceptance criteria for the pancreas donors were age 18–65 years, beating heart and declared brain dead, a known cause of death, negativity for HIV, HBV, HCV, anti-HBc, VDRL-TPHA, absence of risk factors, systemic infections and diabetes, amylase levels within three times the normal range. Tissues were maintained in 0.9% NaCl (Fresenius Kabi, MAH 035725035, Bad Homburg vor der Höhe, Germany) with 10% (v/v) human serum albumin (Kedrion, MAH 022515163, Barga, Italy) solution and were transported from the collection site to the hPO manipulation site in a dedicated shipper (B Medical System, MT 4B, Luxembourg) at 21 ± 4 °C with a temperature detection device (data logger; Testo SE & Co. KGaA, 174 T, Settimo M.se, Italy) according to the manufacturer’s instructions. Islet-depleted tissue remaining after islet isolation (raw material) was processed for hPO isolation within 36 h of initial processing. Quality control (QC) steps, including data log download for evaluation of temperature maintenance during transport and transport document review, were performed soon after delivery.

### Pancreas organoid isolation and culture

Human adult islet-depleted pancreatic tissue was transferred under a laminar hood. A 1-ml sample was taken for sterility testing by the validated GMP bact-Alert method (bioMérieux, Marcy-l’Étoile, France) [26] and 1-ml was dissociated to a single cell suspension, as described below, for testing the viability of the raw material by flow cytometry. Subsequently, raw material was digested in Liberase MTF C/T (0.125 mg/ml, Roche, ref. 5339880001, Basel, Switzerland), neutral protease (0.125 mg/ml, Serva ref. 30303.01, Heidelberg, Germany), and DNase I (0.1 mg/ml, Roche, ref. 3724751103) in human complete medium [27] (see below) at 37 °C for ≤ 2 h or mechanically dissociated by a GentleMACS™ dissociator (Miltenyi Biotec, Teterow, Germany) with the software program m_spleen04 (4×). Whole or duct-enriched pancreatic tissue fragments were embedded in basement membrane extract type 2 (BME2; Trevigen, ref. 3533-005-02, Gaithersburg, MD) and cultured in human complete [AdDMEM/F12 medium (Life Technologies ref. 12634-010, Monza, Italy) supplemented with HEPES (1×, Life Technologies ref. 15630-056, Monza, Italy), Glutamax (1×, Life Technologies ref. 35050-038, Monza, Italy), B27 minus Vitamin A (1×, Life Technologies ref. 12587-010, Monza, Italy), N2 supplement (1×, Life Technologies ref. 17502-048, Monza, Italy) N-acetyl-L-cysteine (1 mM, Sigma-Aldrich ref. A9165, Milan, Italy), R-spondin-1 (RSPO1) recombinant protein (1 μg/ml, Peprotech ref. 120-38. London, UK) or RSPO1 conditioned medium, Noggin recombinant protein (0.1 μg/ml, Peprotech ref. 120-10C, London, UK), epidermal growth factor (EGF, 50 ng/ml, Peprotech ref. AF-100-15, London, UK), gastrin (10 nM, Sigma-Aldrich ref. G9145, Milan, Italy), fibroblast growth factor 10 (FGF10, 100 ng/ml, Prepotech ref. 100-26, London, UK), nicotinamide (10 mM, Sigma-Aldrich ref. N0636, Milan, Italy), forskolin (10 μM, Tocris Bioscience ref. 1099, Bristol, UK), PGE-2 (3 μM, Tocris Bioscience ref. 2296, Bristol, UK), and A83-01 (0.5 μM, Tocris Bioscience ref. 2939, Bristol, UK)]. Where possible, all the research-grade components have been replaced using GMP or clinical-grade ones. All the equipment used in the hPO production was validated in accordance with GMP standards (Installation Qualification, Operational Qualification and Performance Qualification). After isolation, the medium was changed every 3–4 days and hPOs were split every 7 days. Human RSPO1 was purchased (1 μg/ml, R&D System, ref. 4645-RS-01 M, Minneapolis, MN) and RSPO1 CM was produced as previously described [23].

### Cryopreservation

hPOs were collected and mechanically dissociated into fragments. Then ~ 200,000 cells were re-suspended in solution containing 0.9% NaCl (Fresenius Kabi MAH 035725035, Bad Homburg vor der Höhe, Germany) with 10% (volume) dimethyl sulfoxide (Mylan, ref. 67457-178-50, Canonsburg, PA), and 10% (volume) human serum albumin (Kedrion, MAH 022515163, Barga, Italy). Suspensions were cryopreserved in a validated controlled-rate Planer Kryo 360-3.3 freezer (Planer Products PLC, Middlesex, UK). Organoids were stored in liquid nitrogen up to 6 months before thawing experiments.

### In vitro growth curve

At the end of each passage, hPOs were dissociated by trypsinization with 0.5 ml Tryple (Life Technologies ref. 12604013, Monza, Italy) for 5 min. Single-cell suspensions were counted directly with a GMP-validated Nucleocounter® NC-100™ (ChemoMetec, Allerod, Denmark) as previously described [28]. Twenty thousand cells were embedded in a drop of 50 μl BME2, cultured with expansion medium, counted, and reseeded every 2 weeks.

### Karyotype analysis

hPO fragments from confluent cultures were split at a 1:8 ratio and allowed to grow for 4 days and then processed for metaphase analysis. Conventional karyotyping was carried out by Q-banding according to standard laboratory protocols. Numerical and structural abnormalities were analyzed at the 400 banding level, according to the international system for human cytogenomic nomenclature and the European general guidelines and quality assurance for cytogenetics. The analysis included a minimum of 20 metaphase-arrested cells to exclude clonal rearrangements. Metaphase image capture was performed by an automated sample analysis system IKAROS (MetaSystems, Milan, Italy).

### Diameter and area measurement

Bright-field images were acquired with a Nikon Eclipse TS100 microscope equipped with a digital camera (Nikon Instrument Europe). hPOs (*n* = 10 fields) were analyzed at × 4 magnification. At least three independent experiments were performed from P3 to P5. Organoid diameter (major axis) and area (region of interest) were calculated in ImageJ software (https://imagej.nih.gov/ij/).

### Glucose consumption and lactate production measurements in hPO cultures

Glucose and lactate levels were measured with StatStrip® meters [Nova Biomedicals, ref. 47289 (glucose) and ref. 47486 (lactate), Waltham, MA]. The amounts of glucose consumed and lactate produced by hPOs were calculated considering initial glucose and lactate concentrations, respectively, in fresh medium. Three independent measurements of each kind were performed in passages (P) 3–P5.

### Flow cytometry

hPOs and raw material were dissociated by trypsinization with Tryple for 5 min to obtain a single-cell suspension, and 100,000 cells per tube were stained for each sample. The cells were centrifuged for 7 min at 350×*g* and the supernatant was discarded. For surface marker analysis, the pelleted cells were incubated with fluorophore-conjugated antibodies in a total volume of 200 μL of phosphate-buffered saline (PBS, Thermo Fisher Scientific, ref. 14190-094, Waltham, Massachusetts) for 20 min in the dark at room temperature (RT), washed with 3 ml of PBS and resuspended in 200 μL of PBS for analysis. For intracellular marker analysis, the pelleted cells were fixed in 200 μL of 0.25% paraformaldehyde (Electron Microscopy Sciences, ref. 15710, Hatfield, PA) for 1 h at RT, washed with 3 ml of PBS 1% fetal bovine serum (Thermo Fisher Scientific, ref. 10270-106) and permeabilized with 200 μL of Perm Buffer III (Becton Dickinson, ref. 558050, Franklin Lakes, NJ) following manufacturer’s instructions. Next, fixed and permeabilized cells were stained and prepared for analysis as described above. Monoclonal antibodies used in the phenotype analysis are listed in Additional file 1: Table S2. The samples were analyzed in a FACSCanto II cytometer with FACSDiva analysis software (Becton Dickinson). Acquired events were plotted against forwarding scatter-height and forward scatter-area to exclude cell doublets (P1 gate) and then against forward and side scatter-area physical parameters to select homogenous cell populations excluding debris (P2 gate). At least 10,000 P2 events were acquired. Cell viability was assessed with 7-aminoactinomycin D staining (Becton Dickinson). Markers of interest were measured in histograms or dot plots by an analytical gate (P3) for marker-positive events.

### RNA extraction and analysis

Total RNA was extracted from hPOs with TRIzol reagent (Ambion, ref. 15596026, Huntingdon, UK), quantified, and quality checked by NanoDrop ND-1000 spectrophotometry (NanoDrop Technologies, Wilmington, DE). For the qRT-PCR assay, cDNA was synthesized from 500 ng of total RNA with SuperScript IV VILO (Invitrogen, ref. 11756500, Carlsbad, CA). The resulting cDNA was diluted 1:10 and 1 μl was used as a template for quantitative reverse transcription-polymerase chain reaction (qRT-PCR) analysis with PowerUp SYBR Green Master Mix (Applied Biosystems, ref. A15780, Foster City, CA) in a CFX96 thermal cycler (BioRad, Hercules, CA). For each primer pair (Additional file 1: Table S3), amplification efficiency was evaluated according to MIQE guidelines [29]. Relative gene expression levels were determined with the ΔΔCt method; data were normalized to geometric means of endogenous *ACTB* and *TBP* mRNA levels.

### Immunofluorescence analysis

Before the immunofluorescent staining, hPOs were briefly washed with PBS and fixed with 4% PFA (Electron Microscopy Science, ref. 15,710, Hatfield, PA) in PBS for 40 min on ice. Afterward, they were washed with PBS before the rest of the BME2 was removed with Cell Recovery Solution (Corning, ref. 354253, Corning, NY). Next, hPOs were permeabilized with 0.3% Triton X-100 in PBS for 40 min at RT, washed again and incubated three times for 10 min with 100 mM glycine in PBS at RT. After another wash, they were blocked for 12 h at RT with blocking solution [0.1% BSA (Sigma-Aldrich, ref. A8327, Milan, Italy), 0.2% Triton X-100 (Sigma-Aldrich, ref. T8787, Milan, Italy), 0.05% Tween-20 (Sigma-Aldrich, ref. P7949, Milan, Italy) and 10% goat serum (Life Technologies ref. 16210-064, Monza, Italy) in PBS]. The samples were incubated with the primary antibodies diluted in blocking solution for 24 h at 37 °C in a shaker. They were then washed carefully with PBS and incubated with the secondary antibodies and DAPI (Thermo Fisher; 1 μg/ml in PBS) for 2 h at 27 °C. The samples were briefly washed and transferred to a 96-well plate before capturing images with a microscope (Zeiss LSM780).

### Statistical analyses

The results are expressed as mean ± standard error of mean (SEM). Diameter, area, and flow cytometry data were subjected to two-way analyses of variance (ANOVAs) followed by Newman-Keuls post hoc tests for multiple comparisons. Gene expression data were subjected to Student’s *t* tests. All statistical analyses were performed in Prism software version 4.0 (GraphPad, San Diego, CA). *P*-values < 0.05 were considered significant.

## Results

### Validation of tissue transport

Three collection site-to-processing site tissue transport validation runs confirmed compliance with the recommended temperature range (mean, 22.33 ± 0.81 °C; minimum, 19.50 °C; and maximum, 24.00 °C). All five batches were confirmed to be sterile in a BacT/Alert three-dimensional culture system.

### Isolation of hPOs

**Fig. 1:**
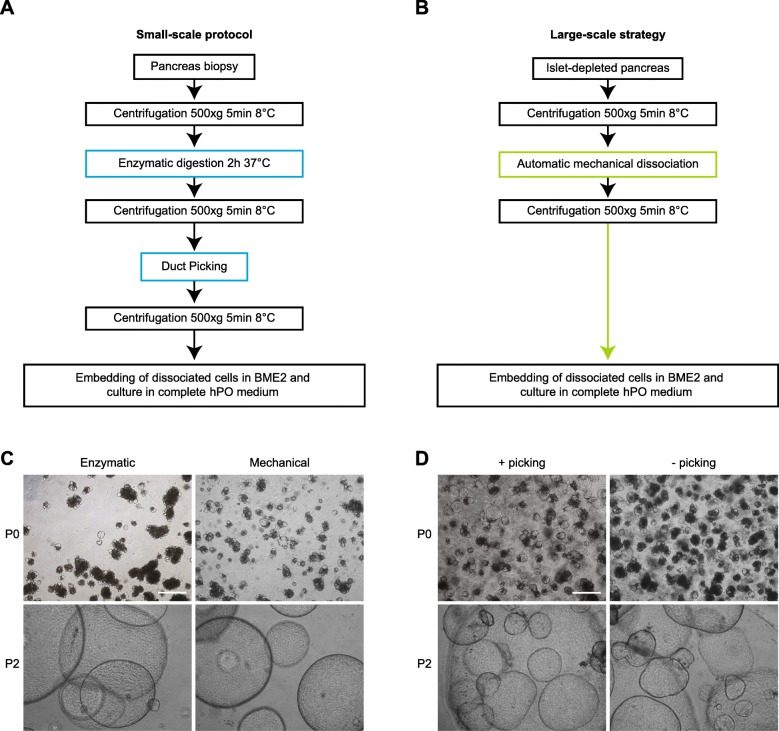
hPO isolation. **a**, **b** Schematic representation of hPO isolation from human adult islet-depleted pancreatic tissue collected to hPO generation. **c** Representative bright-field images of hPOs obtained by enzymatic digestion (left) or mechanical dissociation (right) at P0 and P2. Scale bar, 500 μm. **d** Representative bright-field images of hPOs obtained by mechanical dissociation followed (left) or not (right) by duct picking at P0 and P2. Scale bar, 500 μm

To set up a large-scale strategy for GMP-compliant hPO production, we aimed to adapt the Huch protocol [23] to generate hPOs under GMP grade conditions. For that, it was required to address critical research-grade small-scale protocol steps commonly used for organoid isolation (Fig. 1a). We based our isolation strategy on the use of islet-depleted pancreas rather than pancreas biopsy as a starting material, according to Loomans et al. [8]. Islet-depleted pancreas is an abundant source discarded after clinical isolation of pancreatic islets, and therefore amenable to obtaining large hPO quantities. We processed pancreatic tissues from five donors (Additional file 1: Table S1). Before isolation, the raw material showed a mean viability of 98.82 ± 1.19%. To make the process GMP-compliant, we omitted enzymatic digestion and operator-dependent manual duct fragment picking (Fig. 1b). We split the raw material and performed two hPO isolation strategies in parallel, comparing classical enzymatic digestion to mechanical dissociation. After 5 days, hPOs appeared in cultures and they grew without morphological differences between the two isolation protocols (Fig. 1c).

Next, in another set of experiments, we performed hPO isolation from raw material with and without the manual picking step. Without duct picking, at P0 we observed a mixture of translucent hPO-like structures and of other non-optically transparent cell aggregates, whereas at P2 only hPOs with typical round morphology were obtained (Fig. 1d).

To confirm that hPOs obtained from large-scale protocol were identical with hPOs produced from small-scale protocol, we performed a gene expression analysis. Markers of ductal, acinar, mesenchymal, and endothelial compartments confirmed that hPOs obtained from both protocols were identical (Additional file 1: Figure S1a). All these results were summarized in Additional file 1: Table S4.

These results show that enzymatic digestion can be replaced by mechanical dissociation, and that enrichment picking is not necessary to obtain an hPO culture with typical round morphology. The efficiency of hPO culture establishment was 100%.

### Characterization of hPOs

**Fig. 2:**
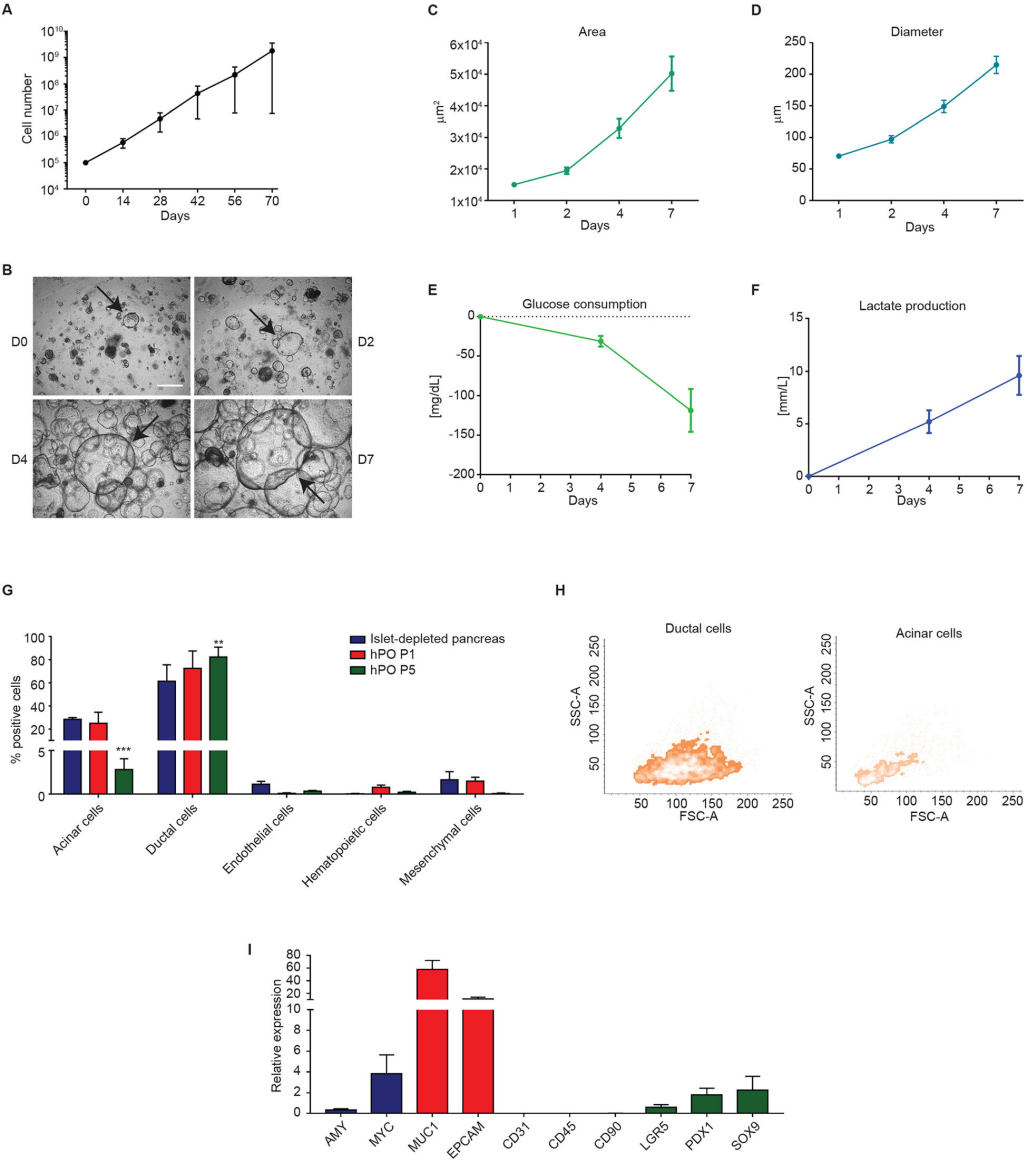
hPO characterization. **a** Growth curve of hPOs; data are means ± SEM (*n* = 4/time point; right panel). **b** Representative bright-field images of hPOs from day 0 to day 7. Scale bar, 500 μm. **c**, **d** Mean (±SEM) diameter and area in a single passage (days 1, 2, 4, and 7). At least 100 organoids were measured. **e**, **f** Mean (±SEM) glucose consumption and lactate production at study time points in a single passage (*n* = 3). **g** Immunophenotypic analysis of adult islet-depleted pancreas and hPOs at different passages to evaluate ductal, acinar, mesenchymal, hematopoietic and endothelial markers. Data are expressed as mean ± SEM (*n*  =  3/group). Two-way analyses of variance (ANOVAs) followed by Newman-Keuls post hoc tests for multiple comparisons was used. **p < 0.01; ***p < 0.0001. **h** Representative flow cytometry density plots of physical parameters and acinar and ductal cell morphologies at P5. **i** Mean gene (±SEM) expression of ductal, acinar, mesenchymal, hematopoietic, endothelial, and pancreatic progenitor markers in hPOs (*n* = 4)

hPOs could be passaged without morphological changes until P5 (Additional file 1: Figure S1b), when they were cryopreserved for biobanking. Assessment of their exponential growth by automated cell counting for 70 days indicated a doubling time at day 7 of 73.02 h (Fig. 2a). We observed a lesser proliferation rate of cultures seeded from single cells for growth curve generation compared to cultures generated from hPO fragments. Pre-biobanking karyotyping of hPOs at P5 (*n* = 5) indicated normal chromosome stability (Additional file 1: Figure S1c). Within a passage, hPOs increased in size (Fig. 2b). Measurement of hPO diameters and areas across different time points in image analysis software indicated that mean hPO diameter and area increased three- and eight-fold, respectively, in 7 days (Fig. 2c, d).

Assessment of culture quality and hPO growth based on quantification of glucose consumption and lactate production in the medium indicated that 31.3 ± 6.84 mg/dL and 118.7 ± 27.14 mg/dL of glucose was consumed after 4 days and 7 days of culture, respectively. The percentages of glucose consumption at day 4 and at day 7 were 13 ± 5% and 49.7 ± 19.7%, respectively. Lactate production was 5.2 ± 1.08 mmol/L at day 4 and increased to 9.6 ± 1.8 mmol/L at day 7 (Fig. 2e, f).

Flow cytometry analysis of islet-depleted pancreatic tissue and hPOs showed a drastic change in the composition of cell populations. First of all, based on the morphology (FSC and SSC physical parameters) the raw material appeared composed by a mix of different populations (Additional file 1: Figure S2a). Distinct cell types were identified based on expression of acinar, ductal, endothelial, hematopoietic, and mesenchymal/stromal markers. This mixed composition with very heterogeneous morphology in the raw material decreased in hPO cultures by P1 and further decreased by P5 (Fig. 2g). The most represented antigens were α-L-fucose glycoprotein (28.3 ± 1.59%), expressed by acinar cells, and SOX9 (61.4 ± 24.7%), expressed by ductal cells. We did not see the relevant percentages of endothelial or mesenchymal stromal cells. The hematopoietic compartment was absent.

In the cultured hPOs, α-L-fucose glycoprotein was stable at P1 but reduced at P5, whereas SOX9 was stable at both P1 and P5. The mesenchymal compartment was markedly reduced at P5 (Fig. 2g). Similarly to the raw material, the two major populations represented in hPO cultures were the acinar and ductal compartments (Additional file 1: Figure S2b). The ductal compartment was highly represented in hPO cultures with larger cells than the acinar compartment (Fig. 2h). Immunofluorescence analysis confirmed that hPOs were mainly composed of ductal cells (SOX9^+^) (Additional file 1: Figure S2c).

To deepen the characterization of cultured hPOs, the intracellular expression of pancreatic markers SOX9 and PDX1 was also addressed. The cultured hPOs showed a consistent amount of PDX1^+^/SOX9^+^cells, whose coexpression indicates the presence of a pancreatic progenitor subpopulation (Additional file 1: Figure S2d).

Molecular analysis of cell-type markers in ductal, acinar, mesenchymal, and endothelial compartments confirmed our immunophenotypic analysis results (Fig. 2i). Taking together, these data demonstrate that hPOs resemble the exocrine pancreas compartments.

### Replacement of RSPO1 CM with the RSPO1 molecule

**Fig. 3:**
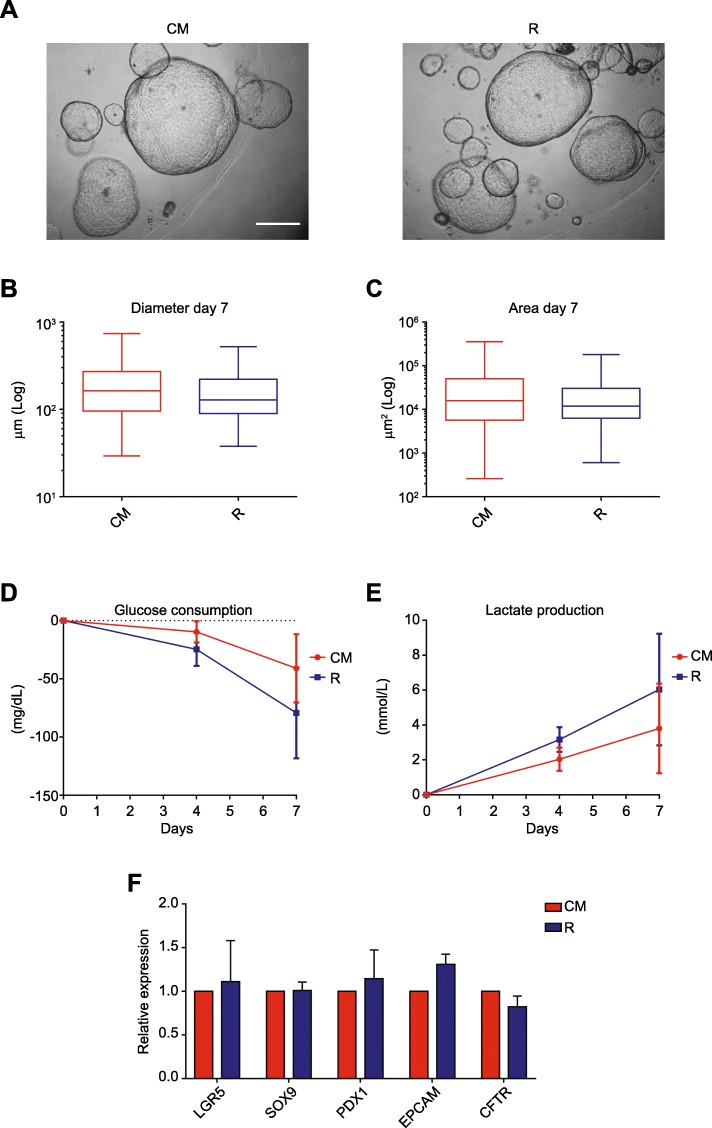
Comparison of RSPO1 CM and RSPO1 molecule cultures. **a** Representative bright-field images of hPOs in culture with RSPO1 CM and R (RSPO1 recombinant molecule). Scale bar, 500 μm. **b**, **c** Mean (±SEM) diameter and area at day 7 in each culture condition (≥ 100 organoids measured) Two-way analysis of variance (ANOVAs) followed by Newman-Keuls post hoc tests for multiple comparisons was used. **d**, **e** Mean (±SEM) glucose consumption and lactate production of hPOs in culture with CM and R at different time points (*n* = 3). Two-way analysis of variance (ANOVAs) followed by Newman-Keuls post hoc tests for multiple comparisons was used. **f** Gene expression analysis of pancreatic markers. Mean expression values (±SEM) were normalized to those of hPOs in CM (*n* = 3) and Student’s *t* tests was used

To obtain a GMP-compliant cellular product, it is recommended that the expansion medium has a well-defined composition. Risk assessment analysis following failure mode effects analysis revealed that RPSO1 CM was straining GMP compliance; it was obtained from an HA-R-Spondin-1293 T transfected cell line and its composition is not defined. In view of replacing it, experiments comparing RPSO1 CM with RSPO1 molecule-supplemented medium (R) were performed (Fig. 3a). hPO diameter and area did not differ significantly between the CM and R cultures across multiple time points after seeding (data not shown). After 7 days in culture, hPOs in CM and R reached confluence and their diameters and areas were similar (Fig. 3b, c).

With respect to metabolic parameters before expansion medium change, glucose consumption 4 days after seeding was 9.67 ± 5.24 mg/dL in CM cultures and 24.67 ± 8.19 mg/dL in R cultures (respective percentages of glucose consumption, 4.34 ± 4.07% and 10.32 ± 5.93%). After 7 days, hPOs in CM had an average glucose consumption of 41.00 ± 16.92 mg/dL and hPOs in R had an average glucose consumption of 79.33 ± 22.52 mg/dL (respective percentages of glucose consumption, 18.39 ± 13.14% and 33.19 ± 16.32%). On the other hand, the average lactate production after 4 days was 2.03 ± 0.38 mmol/L in CM cultures and 3.17 ± 0.41 mmol/L in R cultures, increasing after 7 days to 3.8 ± 1.48 mmol/L and 6.03 ± 1.85 mmol/L, respectively. These biochemical parameters did not differ significantly between the CM and R groups at either time point (Fig. 3d, e). Therefore, R was used for all subsequent experiments in this study. Finally, qRT-PCR indicated that gene expression of selected markers did not differ significantly between the CM and R cultures (Fig. 3f). All these results were summarized in Additional file 1: Table S4.

### Validation of GMP-compliant hPO freezing procedure

**Fig. 4:**
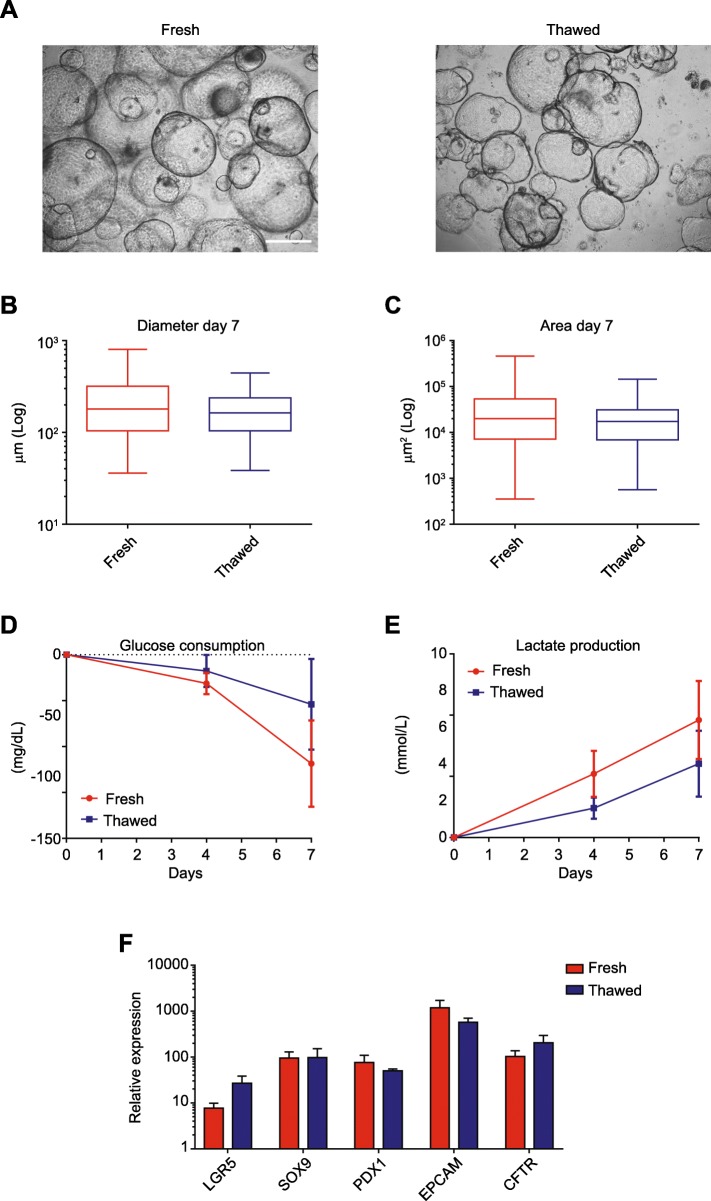
Characterization of hPOs after cryopreservation. **a** Representative bright-field images of fresh hPOs (fresh) and hPOs after thawing (thawed). Scale bar, 500 μm. **b**, **c** Mean (±SEM) diameter and area of fresh and thawed hPOs at day 7 (≥ 100 organoids measured). Two-way analysis of variance (ANOVAs) followed by Newman-Keuls post hoc tests for multiple comparisons was used. **d**, **e** Mean (±SEM) glucose consumption and lactate production of fresh and thawed hPOs at study time points (*n* = 3). Two-way analysis of variance (ANOVAs) followed by Newman-Keuls post hoc tests for multiple comparisons was used. **f** Mean (±SEM) expression of pancreatic markers (*n* = 3) and Student’s *t* tests was used

hPOs were frozen in a controlled-rate freezing machine to minimize the exothermic effects of phase transition and to improve hPO viability after cryopreservation (representative cryopreservation curve in Additional file 1: Figure S3). After thawing, the hPOs had an average viability of 76.67 ± 0.02% measured by Nucleocounter® NC-100™. After freezing and thawing, cultured hPOs showed classical morphology (Fig. 4a). Diameter and area measurements taken after seeding to quantify hPO growth before (fresh) and after (thawed) the freezing process showed no statistically significant differences between the two conditions on day 7 (Fig. 4b, c). Likewise, no significant differences in glucose uptake or lactate production were observed between fresh and thawed hPOs at the days 4 and 7 time points (Fig. 4d, e). Gene profile analysis showed that the gene expression of selected markers did not differ significantly between fresh hPOs and thawed hPOs (Fig. 4f). All these results were summarized in Additional file 1: Table S4.

## Discussion

Diabetes is a common, multisystem disease that results in hyperglycemia due to a relative or absolute insulin deficiency. Currently, two therapeutic options are available for type 1 diabetes. The most common clinical approach for achieving good glycemic control is multiple daily injections of insulin. The second therapeutic option is whole-organ or pancreatic-islet transplantation. Pancreatic islet transplantation is a minimally invasive procedure that can restore normoglycemia and insulin independence in type 1 diabetes, without the surgical complications associated with vascularized pancreas transplantation. Although advances over the past decade have improved patient outcomes dramatically, a number of important issues continue to hamper the success of islet transplantation, including the limited efficiency of the islet isolation process, progressive loss of islet function over time, and the need for multiple donors to achieve insulin independence [22].

The European Community is addressing these limitations in the Horizon 2020 Societal Challenges–Health, demographic change, and well-being pillar, which is supporting the LSFM4LIFE “Production and characterization of endocrine cells derived from human pancreas organoids for the cell-based therapy of type 1 diabetes” project (https://cordis.europa.eu/project/rcn/199753/factsheet/en). In this context, our group is pursuing the possibility of generating a GMP-compliant scaling-up approach to producing hPOs for regenerative medicine applications. Advanced therapy medicinal products (ATMPs) pose several challenges related to intrinsic biological variability, limited stability/shelf life, and incompletely known pharmacodynamics and pharmacokinetics, particularly compared to classic chemical drugs. In this work, we improved a small-scale research protocol for the clinical manufacture of hPO-based ATMPs by implementing large-scale-oriented strategies.

We used islet-depleted pancreas tissue as the starting raw material because it has two major advantages. Firstly, it allows one to obtain a high quantity of undifferentiated organoids at low passages, a reducing manufacturing time and, consequently, ATMP production costs. Because it is leftover tissue from transplantations, it is already suitable for GMP production processes. We decided to use adult endogenous pancreas progenitor cells because they are safer than induced pluripotent stem cell, which facilitates regulatory compliance. The possibility to obtain a large amount of pancreatic progenitor and differentiated derivatives from induced pluripotent stem cells [24] is herein equaled by the implementation of the large-scale protocol starting from adult endogenous pancreas stem cells. Secondly, we made modifications to two critical small-scale research-protocol steps without affecting hPO isolation efficiency or growth: use of automated mechanical dissociation and elimination of the picking procedure. Both changes obviate operator-dependent manipulations that expose the process to contamination risks and impede the speed and efficiency of large-scale undifferentiated hPO production.

Our analysis of expansion-medium origin and formulation indicated that CM collected from RSPO1-expressing cells was the most critical component of the expansion medium that could hamper clinical translation. RSPO1-expressing cells should act as a living chemical factory to produce a highly important growth factor for hPO-based ATMP production consistently. Yet, it would be very challenging to demonstrate that a candidate cell line meets GMP standards for stability, reproducibility, and safety QC standards. We bypassed this challenge by replacing RSPO1 CM with the RSPO1 molecule itself. Even though its use may raise concerns in terms of production costs, such costs would be reduced dramatically in a large-scale GMP setting. In addition, CM production by RSPO1-expressing cells requires the use of bioreactors which have additional costs.

Identity and purity are critical factors in defining and conducting QC of an hPO-based ATMP. Identity, which is defined by phenotypic and/or genotypic features, refers to the confirmation of the presence of the purported bioactive substance. Purity refers to the clinically relevant concentration of that bioactive substance. The particular cellular component that is the key active substance of hPO-based ATMPs has not yet been defined. Thus, our strategy was to characterize an hPO-based ATMP following a standard GMP QC approach. In addition to karyotyping and growth curve analysis, we also obtained an undifferentiated hPO transcriptional profile and immunophenotype, including acinar, ductal, and pancreas progenitor marker expression data. We observed that our undifferentiated hPOs were heterogeneous, containing numerous acinar, ductal, and pancreatic progenitor cells, but no endothelial, mesenchymal, or hematopoietic cells. The ductal cell subset was more substantial than the acinar compartment, likely due to the ductal origin of the hPOs [6]. Interestingly, similar heterogeneity was described previously for human-induced pluripotent stem cell-derived hPOs [24]. These results represent a starting point for setting up product specifications for QC adherence of future hPO-based ATMPs.

Furthermore, we detected a subpopulation of hPO cells that express key pancreatic progenitor markers. The endocrine differentiation potential of hPOs was already described by Loomans et al. [8] upon application of specific differentiation stimuli acting on hPO cells showing progenitor characteristics. On this basis, we can envision a large-scale production of hPO that can be frozen at the undifferentiated stage and subsequently differentiated after thawing to meet specific clinical needs.

Large-scale undifferentiated hPO production in an allogeneic setting will require the use of bioreactors, as have been used with other three-dimensional cellular structures [30]. In this context, we need to maintain cell viability, support growth, minimize cell death, and limit the formation of undesirable metabolites. Thus, in-process controls that will enable real-time monitoring of hPO cultures will need to be introduced into the ATMP manufacturing process, such as glucose consumption and lactate production parameters.

In conclusion, in the present work, we demonstrated the feasibility of our tested approach to GMP-compliant automated generation of undifferentiated hPOs suitable for biobanking and obtained growth parameter data for the hPOs produced. To our knowledge, this work was the first attempt to translate an organoid technology into a GMP-compliant setting for future clinical applications. Further in vivo studies are needed to confirm the ability of organoids to functionally replace an entire organ and to identify therapeutic doses. In the meantime, GMP facilities can demonstrate the feasibility of a standardized large-scale organoid production protocol to generate biobanks of off-the-shelf and ready-to-use undifferentiated hPOs. Hypothetically, in large-scale bioreactor-based culture systems, it should be possible to use a whole islet-depleted pancreas for hPO isolation and expansion. Employing the presently examined strategy, we can infer that approximately 250 × 10^6^ hPOs per islet-depleted pancreas may be available for a regenerative medicine purposes. The present results pave the way for future clinical trials and, ultimately, clinical application of hPOs as a type 1 diabetes therapy.

## Supplementary information

**Additional file 1: **Figure S1. hPO molecular and growth characterization. (a) Gene expression analysis of pancreatic markers. Mean expression values (±SEM) were normalized to those of hPOs obtain from small-scale protocol (*n* = 3) and Student’s t tests was used. (b) Representative images showing the morphology and growth of hPOs from P0 to P5. Scale bar, 500 μm. (c) Representative image of hPO karyotype after five passages in culture. Figure S2. hPO surface and intracellular markers characterization. (a) Representative flow cytometry density plots showing physical parameters and morphology of islet-depleted pancreas tissue and hPOs at different passages. (b) Representative histograms of -Lfucose glycoprotein and SOX9 expression at passage 5. (c) Representative immunofluorescence image of SOX9 in hPOs. Microscope: Carl Zeiss LSM780 confocal microscope. Objective lens: Plan-Apochromat 20x/0.8. Fluorophore Excitation/Emission wavelengths: Dapi (blue): 405/462, Sox9 (violet): 488/562. Scale bar, 100 μm (d) Representative density plots showing unstained (left plot) and stained (right plot) hPOs for detection of PDX1 and SOX9 expression. Histograms showing percentage of PDX1+, SOX9+ and PDX1+/SOX9+ hPO cells (P1; *n* = 10). Figure S3. Controlled rate freezing curve. Representative controlled-rate freezer curve profile for hPO cryopreservation with associated ramp parameters. Table S1. Human islet donor characteristics. Table S2. Antibody list. Table S3. Primer sequences. Table S4. Results of different methods and options for clinical translation

## Data Availability

The datasets supporting the conclusions of this article are included within the article and its additional files.

## Abbreviations

ATMP: Advanced therapy medicinal products
BME2: Basement membrane extract type 2
cDNA: Complementary DNA
GMP: Good manufacturing practices
hPO: Human pancreas organoids
PDX1: Pancreatic and Duodenal Homeobox 1
QC: Quality controls
qRT-PCR: Quantitative reverse transcription-polymerase chain reaction
RSPO1 CM: R-spondin-1 Conditioned Medium
RSPO1 R: R-spondin-1 Recombinant molecule
SEM: Standard error of mean
SOX9: Sex-determining region Y-box 9
