# Supplementary material for: Standardized GMP-compliant scalable production of human pancreas organoids
Source: Stem Cell Res Ther. 2020 Mar 4;11:94. doi: 10.1186/s13287-020-1585-2 (PMC7055108; doi:10.1186/s13287-020-1585-2)
Supplement: Supplementary file 1 — Additional file 1: Figure S1. hPO molecular and growth characterization. (a) Gene expression analysis of pancreatic markers. Mean expression values (±SEM) were normalized to those of hPOs obtain from small-scale protocol (n = 3) and Student’s t tests was used. (b) Representative images showing the morphology and growth of hPOs from P0 to P5. Scale bar, 500 μm. (c) Representative image of hPO karyotype after five passages in culture. Figure S2. hPO surface and intracellular markers characterization. (a) Representative flow cytometry density plots showing physical parameters and morphology of islet-depleted pancreas tissue and hPOs at different passages. (b) Representative histograms of -Lfucose glycoprotein and SOX9 expression at passage 5. (c) Representative immunofluorescence image of SOX9 in hPOs. Microscope: Carl Zeiss LSM780 confocal microscope. Objective lens: Plan-Apochromat 20x/0.8. Fluorophore Excitation/Emission wavelengths: Dapi (blue): 405/462, Sox9 (violet): 488/562. Scale bar, 100 μm (d) Representative density plots showing unstained (left plot) and stained (right plot) hPOs for detection of PDX1 and SOX9 expression. Histograms showing percentage of PDX1+, SOX9+ and PDX1+/SOX9+ hPO cells (P1; n = 10). Figure S3. Controlled rate freezing curve. Representative controlled-rate freezer curve profile for hPO cryopreservation with associated ramp parameters. Table S1. Human islet donor characteristics. Table S2. Antibody list. Table S3. Primer sequences. Table S4. Results of different methods and options for clinical translation [file 13287_2020_1585_MOESM1_ESM.pdf]

Supplementary Figure 1_Dossena et al.

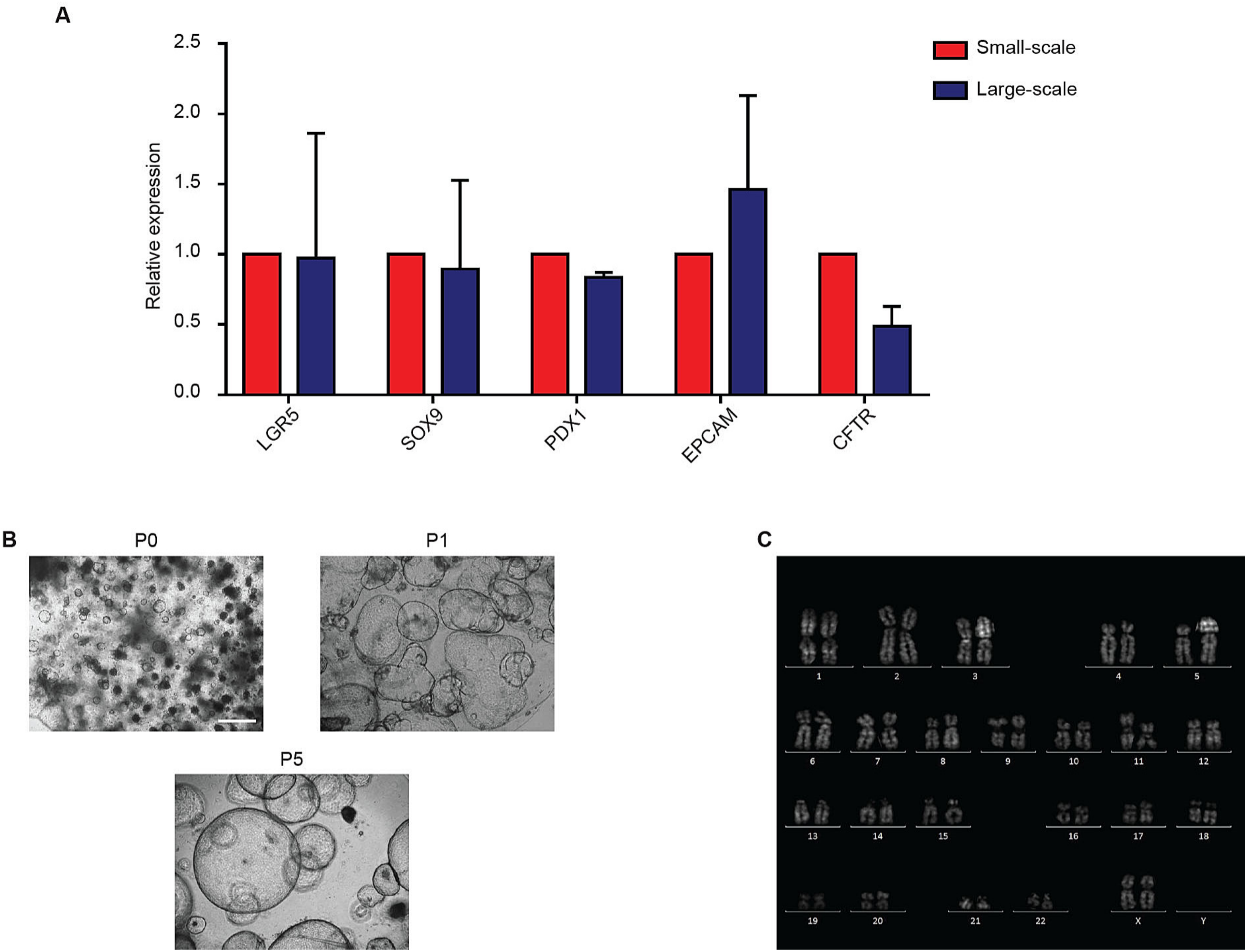

Supplementary Figure 2_Dossena et al.

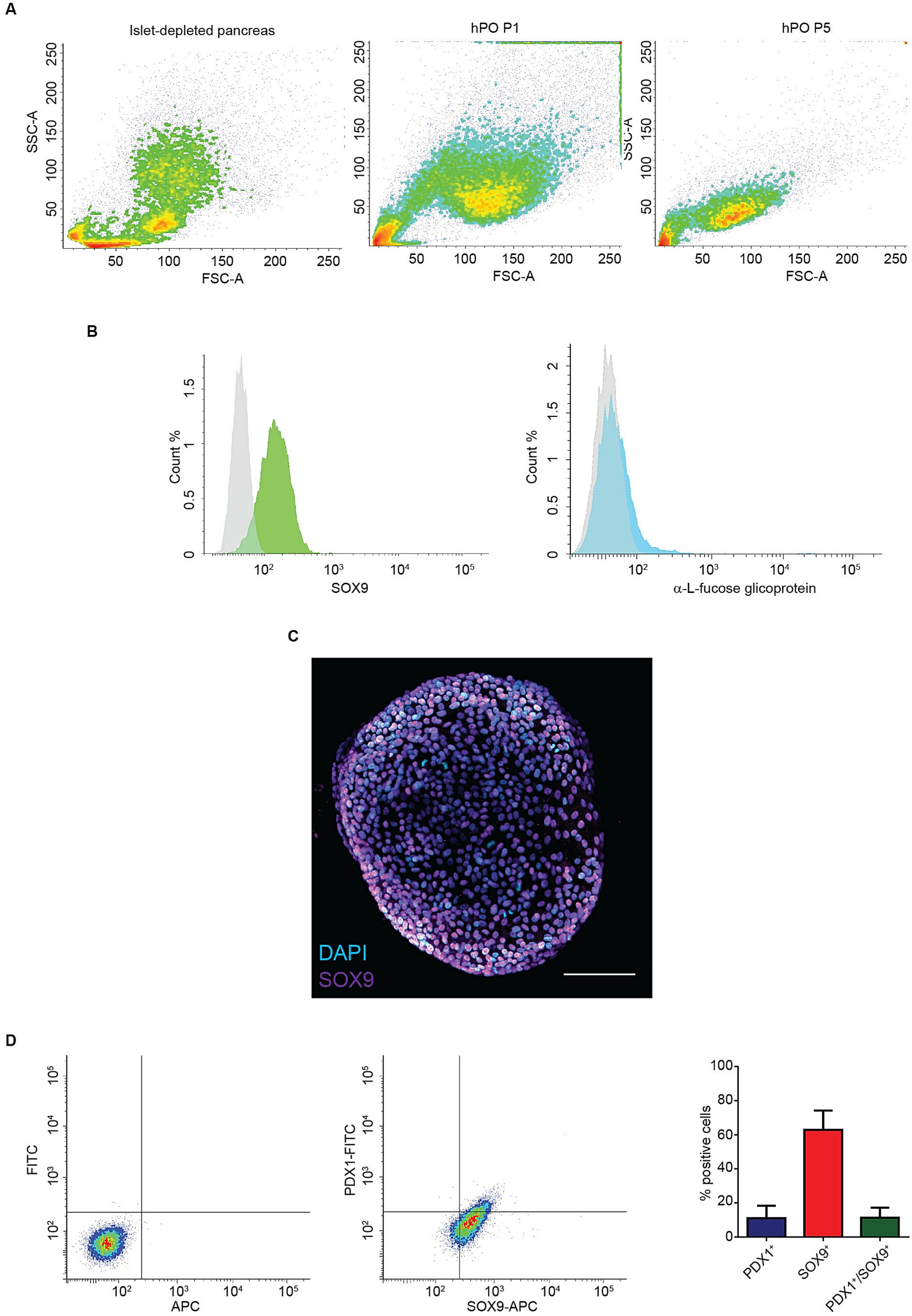

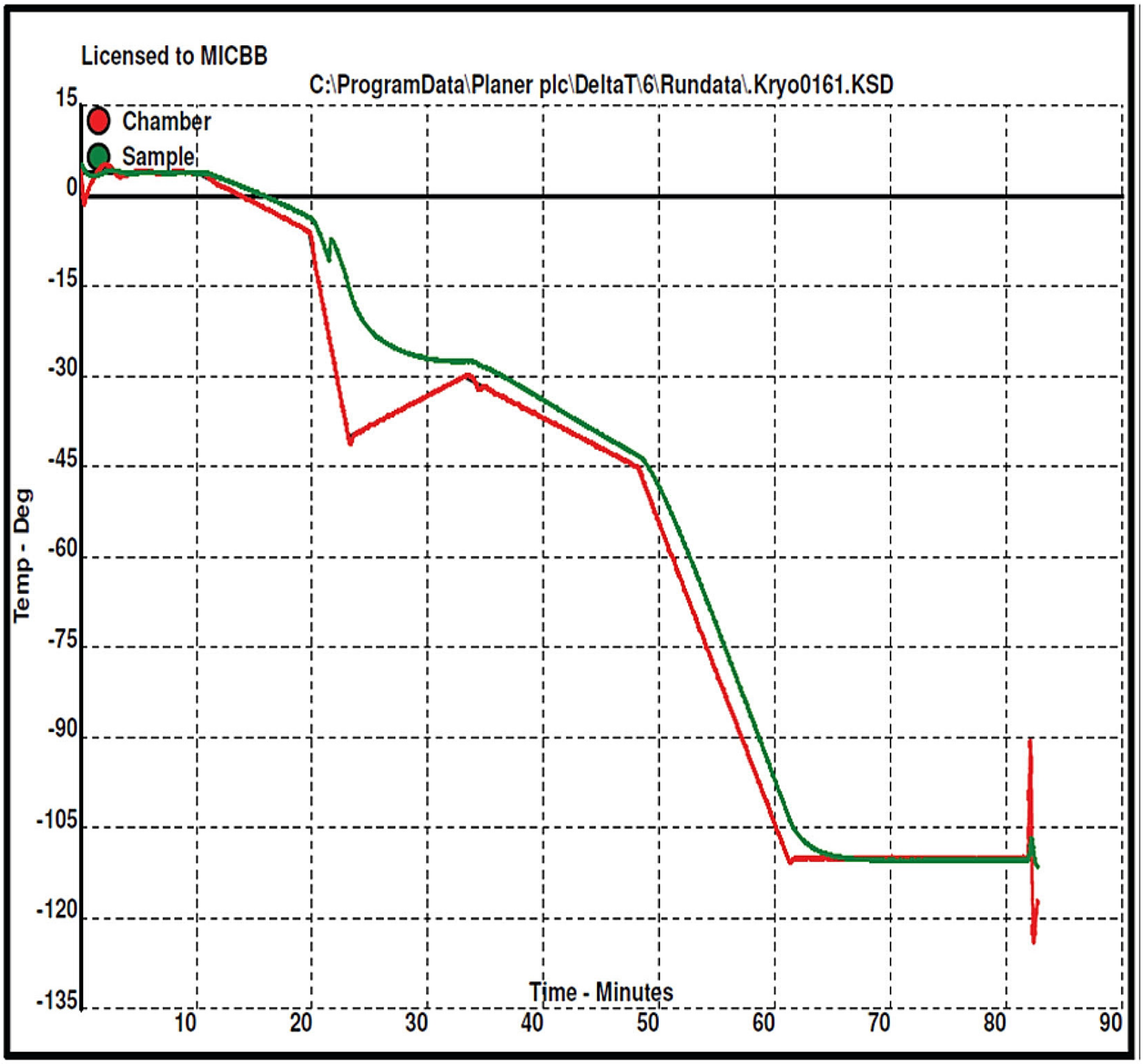

No Start Annotation
KryoFile Start Time : 02-Jan-2018 11:35
Communications Error
No End Annotation
KryoFile End Time : 02-Jan-2018 13:28

Profile :

Date of Last Edit : 02-Jan-2018 13:28
Configuration : C:\ProgramData\Planer plc\DeltaT\6\MRV.KFG
Start Temperature : 4.0
Seeding Type : No Seeding

Step No.	Rate	End Temp	Hold (m s)	Trigger
1			10m 0s	Chamber
2	-1.00	-5.70		Chamber
3	-10.00	-40.00		Chamber
4	1.00	-30.00		Chamber
5	-1.00	-45.00		Chamber
6	-5.00	-110.00		Chamber

Table S1

Human islet donor characteristics

Age (yrs)	Gender	Body mass index
56	Female	21.2
42	Female	19.7
60	Male	26.3
62	Male	23.1
63	Female	25.0

Table S2

Antibody list

**Antigen**	**Detecting molecule**	**Supplier**	**Dilution**	**Reference**
CD90	PC5	Beckman Coulter	1:40	PN IM37036
CD73	APC	Miltenyi Biotec	1:40	130-095-183
CD31	PE	BD Pharmingen	1:40	555446
CD45	APC-H7	BD Pharmingen	1:100	560178
α-L-fucose glycoprotein	FITC-conjugated lectin	Sigma Aldrich	1:2000	L9006
SOX9	Alexa Fluor®647	BD Pharmingen	1:100	565493
PDX1	Alexa Fluor®488	BD Pharmingen	1:100	562274

Table S3

Primer sequences

**Marker for**	**Gene**	**Forward (5’→3’)**	**Reverse (3’→5’)**
Pancreatic progenitors	*LGR5*	ACCAGACTATGCCTTTGGAAAC	TCCCAGGGAGTGGATTCTATT
	PDX1	CACATCCCTGCCCTCCTAC	GAAGAGCCGGCTTCTCTAAAC
	SOX9	GTACCCGCACTTGCACAAC	TCTCGCTCTCGTTCAGAAGTC
Ductal cells	*MUC1*	AGCCACTTCTGCCAACTTGT	TGTCCGAGAAATTGGTGGG
	EpCAM	GCTGGCCGTAAACTGCTTTG	ATCATTGTTCTGGAGGGCCC
	CFTR	CACAGGACAGCCCTTCTTTC	TGCCCATGGCCTATCTACTT
Acinar cells	*MYC*	GCTGCTTAGACGCTGGATTT	TAACGTTGAGGGGCATCG
	AMY	CCTTCTGACAGAGCGCTTGTC	CAGCCCCATGTCCTCGTT
Endothelial cells	*CD31*	ACCACCAGAGAAGGGCAAT	GCACCAGGAGTAGGAATAGGG
Mesenchymal cells	*CD90*	CGAACCAACTTCACCAGCAAAT	CCTTGCTAGTGAAGGCGGATA
Housekeeping genes	*ACTB*	CACGATGGAGGGGAAGACGG	CGCCGCCAGCTCACCATG
	TBP	GCCACGCCAGCTTCGGAGAG	CCGCAGCAAACCGTTGGGA

Table S4

Results of different methods and options for clinical translation

		**Small-scale protocol**	**Large-scale protocol-CM Rspo1**	**Large-scale protocol-R Rspo1**	**After cryopreservation**
**Organoid formation**		100%	100%	100%	100%
**Markers gene expression***	**LGR5**	26.36±3.32	26.35±0.62	26.82±1.88	27.04±0.26
	**SOX9**	24.18±2.37	23.82±1.65	24.18±1.99	25.23±0.44
	**PDX1**	26.61±2.39	26.73±0.53	26.61±0.15	26.06±0.26
	**EpCAM**	21.44±1.06	21.83±0.9	26.61±0.89	22.56±0.27
	**CFTR**	25.27±2.06	25.07±1.01	25.27±1.18	24.10±0.65
**Area day7 (μm^2)**		n.d.	41472±6817	40190.9±5419.7	26757±3399
**Diameter day7 (μm)**		n.d.	202.8±16.14	214.58±13.7	181.8±11.03
**Glucose consumption day7 (mg/dL)**		n.d.	41.00±29.31	118.7±27.14	54±49.43
**Lactate production day7 (mmol/L)**		n.d.	3.8±2.57	9.6±1.8	6.03±2.70

*values expressed as 2-ΔCt and were normalized to geometric means of endogenous ACTB and TBP mRNA levels

Figure S1. hPO molecular and growth characterization.

(a) Gene expression analysis of pancreatic markers. Mean expression values (±SEM) were normalized to those of hPOs obtain from small-scale protocol (n = 3) and Student's *t* tests was used. (b) Representative images showing the morphology and growth of hPOs from P0 to P5. Scale bar, 500 µm. (c) Representative image of hPO karyotype after five passages in culture.

Figure S2. hPO surface and intracellular markers characterization

(a) Representative flow cytometry density plots showing physical parameters and morphology of islet-depleted pancreas tissue and hPOs at different passages. (b) Representative histograms of α-L-fucose glycoprotein and SOX9 expression at passage 5. (c) Representative immunofluorescence image of SOX9 in hPOs. Microscope: Carl Zeiss LSM780 confocal microscope. Objective lens: Plan-Apochromat 20x/0.8. Fluorophore Excitation/Emission wavelengths: Dapi (blue): 405/462, Sox9 (violet): 488/562. Scale bar, 100 µm

(d) Representative density plots showing unstained (left plot) and stained (right plot) hPOs for detection of PDX1 and SOX9 expression. Histograms showing percentage of $PDX1^+$, $SOX9^+$ and $PDX1^+/SOX9^+$ hPO cells (P1; n=10).

Figure S3. Controlled rate freezing curve.

Representative controlled-rate freezer curve profile for hPO cryopreservation with associated ramp parameters.
